# Severe Little Ice Age drought in the midcontinental United States during the Mississippian abandonment of Cahokia

**DOI:** 10.1038/s41598-021-92900-x

**Published:** 2021-07-05

**Authors:** David P. Pompeani, Broxton W. Bird, Jeremy J. Wilson, William P. Gilhooly, Aubrey L. Hillman, Matthew S. Finkenbinder, Mark B. Abbott

**Affiliations:** 1 Department of Geology, Kansas State University, Manhattan, KS 66506 USA; 2 Department of Earth Science, Indiana University-Purdue University, Indianapolis, IN 46202 USA; 3 Department of Anthropology, Indiana University-Purdue University, Indianapolis, IN 46202 USA; 4 Department of Atmospheric and Environmental Sciences, University at Albany, State University of New York, Albany, NY 12222 USA; 5 Department of Environmental Engineering and Earth Science, Wilkes University, Wilkes-Barre, PA 18766 USA; 6 Department of Geology and Environmental Science, University of Pittsburgh, Pittsburgh, PA 15260 USA

**Keywords:** Palaeoclimate, Climate-change impacts, Limnology

## Abstract

Drought has long been suspected as playing an important role in the abandonment of pre-Columbian Native American settlements across the midcontinental United States between 1350 and 1450 CE. However, high-resolution paleoclimatic reconstructions reflecting local effective moisture (the ratio of precipitation to evaporation) that are located in proximity to Mississippi period (1050–1450 CE) population centers are lacking. Here, we present a 1600-year-long decadally resolved oxygen isotope (δ^18^O) record from Horseshoe Lake (Collinsville, IL), an evaporatively influenced oxbow lake that is centrally located within the largest and mostly densely populated series of Mississippian settlements known as Greater Cahokia. A shift to higher δ^18^O in the Horseshoe Lake sediment record from 1200 to 1400 CE indicates that strongly evaporative conditions (i.e., low effective moisture) were persistent during the leadup to Cahokia’s abandonment. These results support the hypothesis that climate, and drought specifically, strongly impacted agriculturally based pre-Columbian Native American cultures in the midcontinental US and highlights the susceptibility of this region, presently a global food production center, to hydroclimate extremes.

## Introduction

**Figure 1:**
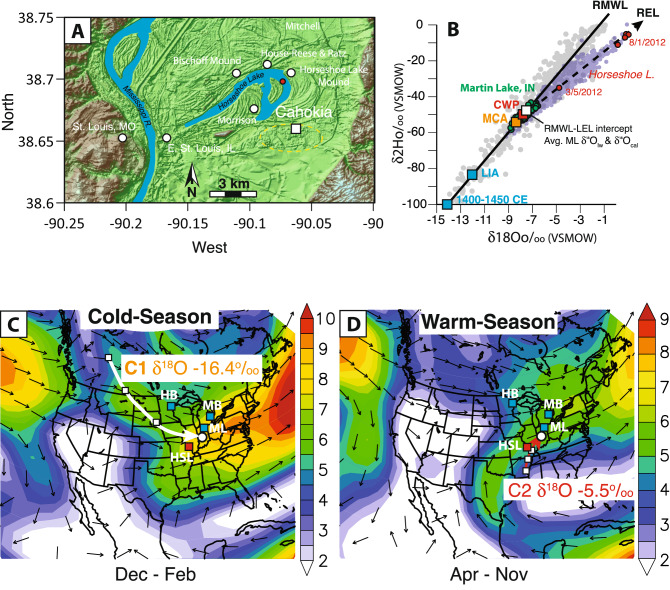
(**A**) Map of the American Bottom portion of the Mississippi River floodplain showing Horseshoe Lake’s position relative to prominent Mississippian urban centers (white circles), the largest of which was Cahokia (yellow dashed circle). Monks Mound, the largest earthwork at Cahokia, is indicated with a white square. The location from which the sediment core was collected is indicated with a red circle. (**B**) Hydrogen and oxygen isotope results for Indianapolis, IN, precipitation events (gray circles), Martin Lake, IN (green circles), Midwest lakes (light blue circles), and Horseshoe Lake (red circles). The white box indicates the intersection between the REL and RMWL that defines regional annual average δ^18^O_precip_. Average isotopic compositions of precipitation during past climate events, including the Medieval Climate Anomaly pluvial (orange square), Little Ice Age average and most severe drought from 1350 to 1450 CE (blue squares), and current warm period (red square) are also shown. δ^18^O values were calculated from δ^18^O_cal_ using an average temperature of 18 °C. Composite mean maps of (**C**) cold-season (December–March) and (**D**) warm-season (April–November) 850 hPa vector winds between 1949 and 2015 CE are from the NCEP-NCAR reanalysis database^13^. Arrows are wind directions with colored shading indicating velocity (m/s). Also plotted are mean back trajectories for cluster 1 (thick white line in **A**) and cluster 2 (thick red line in **B**) of the event-based Indianapolis, IN, precipitation samples. White squares are spaced at 24-h intervals. White circles indicate the starting point from which back trajectories were calculated. The mean back trajectories are in agreement with their respective seasonal atmospheric circulation patterns since 1949 CE. Paleoclimate records discussed in the text are shown with blue squares. Horseshoe Lake is indicated with a red square. Images provided by the NOAA/ESRL Physical Sciences Division, Boulder Colorado (http://www.esrl.noaa.gov/psd/).

Between 1050 and 1450 CE, pre-Columbian (pre-1492 CE) Native American agriculturalists, collectively referred to as the Mississippians, developed and occupied an extensive network of settlements with large earthen mounds and plazas across the central Mississippi and lower Ohio River valleys^1^. The largest of these, Cahokia, was located near present-day Collinsville, IL (Fig. 1) and had an estimated peak population of 15,000–20,000 from 1100 to 1200 CE^2,3^. The Mississippians at Cahokia constructed plazas, causeways, fortifications, and hundreds of monumental earthen mounds, including Monks Mound^4^, which is the largest pre-Columbian North American earthwork north of Mexico^5,6^. Cahokia, and the associated St. Louis, East St. Louis and Mitchell precincts (i.e., Greater Cahokia), experienced rapid growth during this time, with locals and immigrants also populating the surrounding uplands to the east in a series of sites referred to as the Richland Complex^7^. After 1200 CE, social change and population decentralization occurred at Cahokia, as well as the surrounding regions^8^. This includes archaeological evidence for the construction of palisade walls around Cahokia’s central precinct^9^ and skeletal trauma patterns on human remains from the broader region indicating elevated levels of interpersonal violence and warfare^9,10^. Midcontinental population centers were ultimately abandoned by ca. 1450 CE, with only intermittent or small-scale occupations thereafter^11,12^.

While the reasons behind the sudden growth and decline of the Mississippians are debated, climate change likely played a central role given that (1) Mississippian societies were dependent on agriculture and hence susceptible to warm-season drought, and (2) that the Vacant Quarter was formed during the Little Ice Age (LIA; ~ 1250–1830 CE), a time of known climatic volatility^14–17^. The role of climate is supported by recent findings that show an especially severe warm-season drought occurred across the midcontinental US between 1350 and 1450 CE as part of generally drier mean state conditions during the LIA^13^. While a climatic influence on the Mississippian period depopulation of the midcontinent is compelling, high-resolution paleoclimate evidence for drought conditions at major Mississippian population centers, like Greater Cahokia, is lacking.

Here, we test the climate hypothesis using a 1600-year-long authigenic calcite oxygen isotope record from Horseshoe Lake (δ^18^O_HSL_), an evaporatively modified oxbow lake situated just north of Cahokia and centrally located within the greater American Bottom region. Because of its sensitivity to evaporation, the δ^18^O_HSL_ record reflects changes in the local balance between precipitation and evaporation (P/E). As such, the Horseshoe Lake record provides a means to assess the timing and nature of P/E balance changes (i.e., pluvial versus drought) that would have directly impacted the agricultural foundations of Cahokia and associated population centers, such as the St. Louis, East St. Louis, and Mitchell precincts^18,19^. The impact of climate change on Greater Cahokia’s occupation is further investigated by comparing the δ^18^O_HSL_ record with published geochemical proxies of anthropogenic disturbances from Horseshoe Lake^20^. When interpreted with previously published proxy records, δ^18^O_HSL_ reveals the temporal correspondence between regional P/E balance variability, Mississippian occupation, and land-use patterns.

## Study site

Horseshoe Lake (38.6983°, − 90.0731°, 126 m asl) is a shallow (~ 5 m), oxbow lake (12 km^2^) with a ca. 200 km^2^ watershed located in the American Bottom portion of the Mississippi River floodplain just below the confluence with the Missouri River (Fig. 1). Although Horseshoe Lake is often flooded, its shallow depth and large surface area results in a hydrology that is sensitive to evaporation. This was reflected in field observations between the spring and summer of 2012 CE^21^, when drought caused lake levels to visibly fall and lake water δ^18^O values increased (Fig. 1B). Despite modifications to the lake’s watershed over the last 150 years, the lake’s residence time today is similar to the past because reductions in inflows as a result of stream diversions were offset by reductions in volume due to increased sedimentation (supplemental information). The modern responses to evaporation are therefore useful for considering how the lake would have responded to past droughts, including during the Mississippi Period. Authigenic calcite precipitated from the lake’s alkaline waters constitute an archive of variations in lake water δ^18^O that respond to changing effective moisture conditions that span the last 1600 years^20^.

Like much of the midcontinental US, climatic conditions in the American Bottom are characterized by strong seasonality. Winters are typically dominated by enhanced ridge and trough atmospheric circulation from November through March, which steers cold and dry ^18^O-depleted airmasses (δ^18^O_precip_ =  − 16.4‰) from the Pacific and Arctic into the region, resulting in precipitation and temperature minimums (Fig. 1)^13,22^. Warm-season conditions, conversely, are predominated by more zonal atmospheric circulation, which enhances clockwise atmospheric circulation over the eastern US and draws warm, moisture-laden air masses with less negative isotopic values (δ^18^O_precip_ =  − 5.5‰) from the Gulf of Mexico into the midcontinent (Fig. 1)^13^. As such, the warm season is characterized by temperature and precipitation maxima^13^. Changes in the frequency of air mass incursions from these two moisture sources, and hence the seasonal distribution and isotopic composition of precipitation, are strongly modulated by the Pacific North American (PNA) teleconnection, especially during the winter^23–25^. During negative (−) PNA phases, zonal atmospheric circulation is strengthened, increasing delivery of moisture from the Gulf of Mexico and resulting in high δ^18^O_precip_ values. Positive (+) PNA phases are characterized by ridge (over the Rocky Mountains) and trough (over the midcontinental US) atmospheric circulation, increasing the frequency of northwesterly air mass incursions with low δ^18^O_precip_ into the midcontinental US^13,26^. On decadal and longer timescales, PNA-like variability is modulated by the Pacific Decadal Oscillation (PDO), in which + PNA conditions are generally associated with + PDO conditions and vice versa^27^.

## Results

Six surface water samples were analyzed from Horseshoe Lake and Mississippi River during March and August of 2012 CE. Surface water recovered from Horseshoe Lake in March were relatively depleted (δ^18^O =  − 4.8‰, δ^2^H =  − 34.7‰), reflecting reduced evaporation during the spring (Fig. 1B). During the severe drought conditions of August^21^, surface water levels visibly fell in Horseshoe Lake and δ^18^O values were enriched to + 1.0‰, an increase of 5.8‰ since March. The isotopic composition of surface water at Horseshoe Lake was more enriched than the isotopic composition of surface water recovered from the Mississippi River (δ^18^O =  − 10.6‰, δ^2^H =  − 84.8‰), demonstrating the lake’s sensitivity to evaporation.

**Figure 2:**
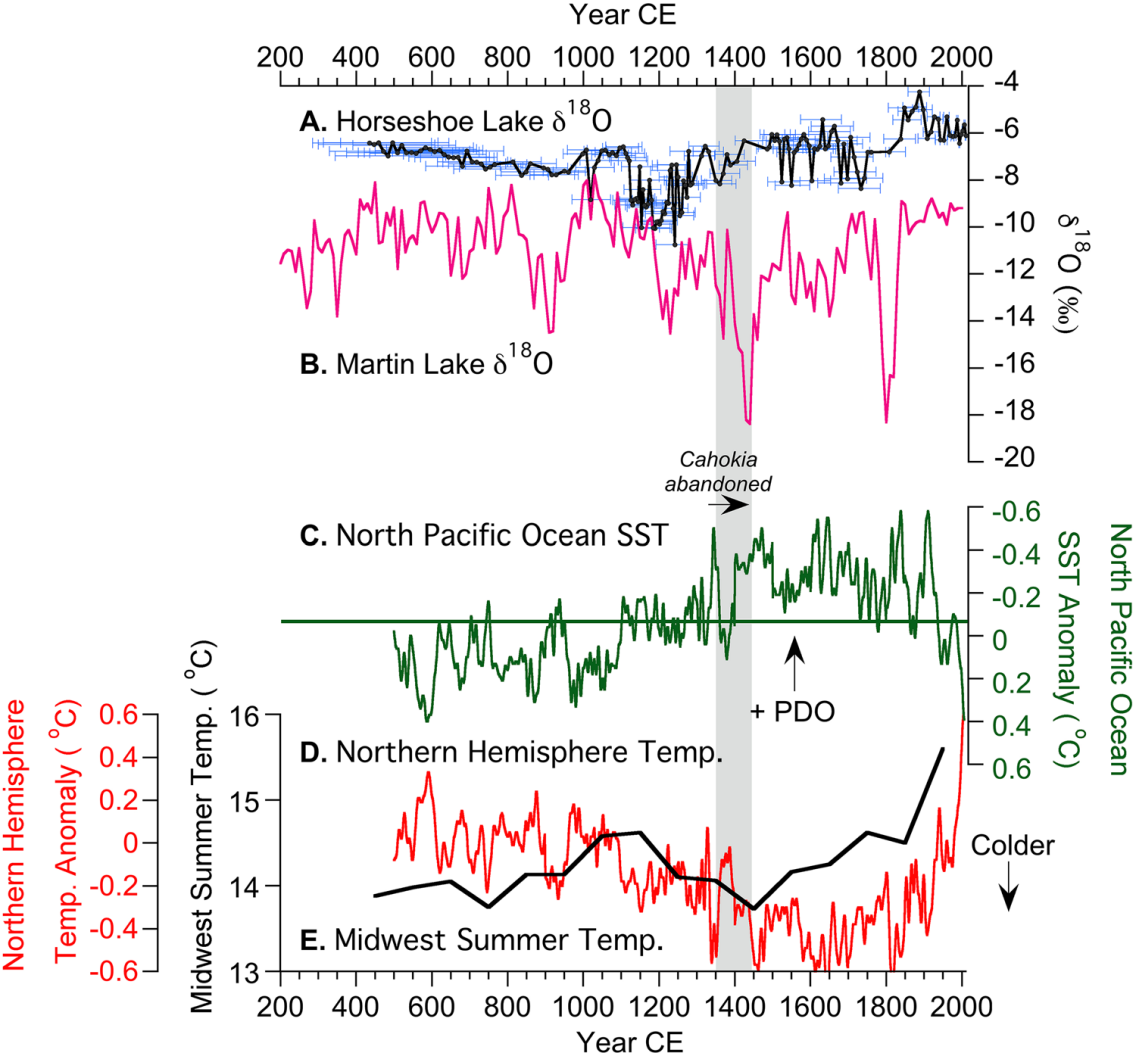
Proxy records for the last 1600 years. (**A**) Horseshoe Lake calcite δ^18^O data with 95% confidence intervals (this study). (**B**) Martin Lake calcite δ^18^O data^13^. (**C**) North Pacific Ocean sea surface temperature (SST) reconstruction. Negative SST anomalies indicate positive Pacific Decadal Oscillation (PDO) conditions^15^. (**D**) Northern hemisphere temperature anomalies^15^ compared against the (**E**) pollen-based Midwest summer temperature reconstruction from Viau, et al.^28^.

Radiometric dates (i.e., ^14^C and ^210^Pb) indicate that Horseshoe Lake’s eastern basin contains calcite-rich sediment spanning at least the last 1600 years^20^, with each δ^18^O_HSL_ measurement representing about 10 years on average (Fig. 2A). δ^18^O_HSL_ values gradually decreased from ca. 400 to 1100 CE. Isotope values then abruptly decreased by 3.5‰ from 1100 to 1200 CE. From 1200 to 1450 CE, δ^18^O_HSL_ increased by 3.7‰ and remained relatively stable from 1450 to 1800 CE. Starting around 1800 CE, δ^18^O_HSL_ increased again reaching maximum values (− 4.2‰) in 1870 CE (Fig. 2A). After 1870 CE, δ^18^O_HSL_ gradually declined until the end of the record in 2012 CE.

## Discussion

These δ^18^O_HSL_ results are similar to a previously published low-resolution δ^18^O record from Horseshoe Lake that spanned the period from 940 to 1860 CE^29^, demonstrating that the δ^18^O_HSL_ signal is robust. White et al.^29^, however, interpreted δ^18^O_HSL_ as reflecting variations in summer versus winter precipitation, which neglects the significant influence of evaporative processes on the lake water (Fig. 1B), and hence δ^18^O_cal_. A 5.8‰ increase in lake water δ^18^O and decline in water levels during the historic drought of 2012 CE^21^ suggests that modern Horseshoe Lake water is extremely sensitive to evaporation, and thus changes in P/E balance. Due to Horseshoe’s large surface area/volume ratio, both today and in the past, evaporation likely had a strong influence on lake water δ^18^O. The observed lake water isotopic enrichment in 2012 CE, for example, cannot be explained by changing precipitation sources or temperature alone because δ^18^O and δ^2^H values plot to the right of the regional meteoric water line on the regional evaporation line (Fig. 1B). This demonstrates that under reduced P/E, a substantial amount of water was removed from Horseshoe Lake via evaporation. Although the present watershed has been altered, water δ^18^O values were likely sensitive to evaporation in the past. For example, a major alteration of Horseshoe’s watershed occurred during the construction of the Cahokia Diversion Channel in the 1920s, which diverted a major portion of stream input away from the lake^30^. This change to watershed hydrology was offset by reductions in accommodation space as a result of sedimentation. As a result, Horseshoe Lake’s residence time remained effectively unchanged (supplemental information). This fact is illustrated by the minimal impact on the δ^18^O_HSL_ record, which shows that the isotopic signal was not impacted by watershed modifications and is instead still mainly controlled by changes in P/E balance. In light of this evidence, we contend that evaporation would have been a significant influence on the isotopic composition of water at Horseshoe Lake in the past (as it is today), and hence δ^18^O_HSL_ is a reliable indicator of past effective moisture (P/E) changes.

In addition to evaporation, the δ^18^O of authigenic calcite can also be affected by variations in temperature. However, estimated temperature changes of + / − 2 °C (see Fig. 2D, E) and historical drought temperatures^31^ are not large enough to explain the full 6.5‰ range in δ^18^O variability observed in the Horseshoe Lake sediment record. Taking into account the effects of temperature on δ^18^O includes a + 0.59‰ °C^−1^ influence on precipitation δ^18^O^32^ and − 0.22‰ °C^−1^ for calcite forming from lake water (i.e., δ^18^O_HSL_)^33^. The combined influences result in approximately 0.36‰ °C^−1^ in response to temperature changes. A 2 °C change during the LIA, for example, would then equate to a 0.72‰ change in δ^18^O_HSL_. For temperature to play a dominant role in δ^18^O_HSL_ variability, past temperature variations would need to be on the order of 18 °C (6.5‰ δ^18^O range), which are unrealistically high (Fig. 2D, E).

Comparisons of δ^18^O_HSL_ with the Martin Lake sediment calcite δ^18^O record (i.e., δ^18^O_ML_) (41.56°, − 85.38°) suggest considerable changes in the predominance of different moisture sources to the midcontinent over the last 1600 years (Fig. 2B). Martin Lake, a small kettle lake in northeastern Indiana, has a short water residence time such that the δ^18^O of its surface waters reflects the isotopic composition of regional meteoric water (Fig. 1B)^13^. Although located approximately 500 km from Horseshoe Lake, the isotopic composition of precipitation at Martin and Horseshoe lakes are controlled by the same synoptic-scale atmospheric circulation processes^13^ and, as such, show consistent seasonal trends and similar annual average values that differ by less than 1‰ (Fig. S2). We therefore use the Martin Lake record to infer regional trends in the isotopic composition of precipitation as controlled by changes in atmospheric circulation during the last 1600 years. Accordingly, high δ^18^O at Martin Lake (δ^18^O_ML_) indicates persistent − PNA-like atmospheric circulation that increased the advection of moisture from the Gulf of Mexico. Conversely, low δ^18^O_ML_ indicates + PNA-like atmospheric circulation patterns characterized by more frequent incursions of isotopically depleted airmasses from the northwest (Fig. 1C).

**Figure 3:**
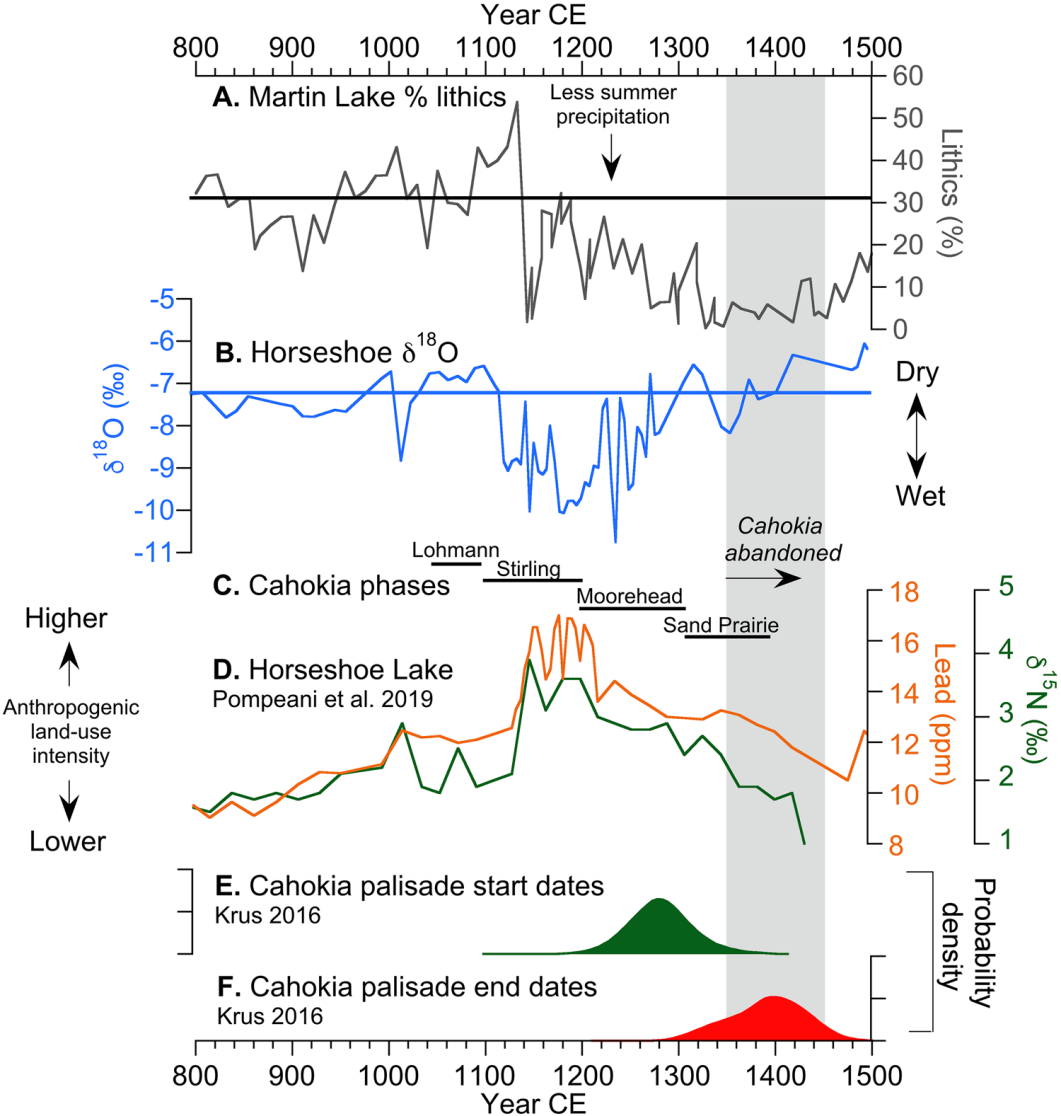
Proxy records from 800 to 1500 CE. (**A**) The Martin Lake % lithic record^13^. (**B**) Horseshoe Lake calcite δ^18^O data (this study). (**C**) Archaeological phases at Cahokia^38^ compared against (**D**) anthropogenic disturbance proxies^20^ and radiocarbon probability density functions for the (**E**) start and (**F**) end of palisade wall construction around Monks Mound^9^.

Lower δ^18^O_HSL_ and higher δ^18^O_ML_ from 400 to 1200 CE indicate relatively high average P/E (i.e., wet conditions) during this time, whereas higher δ^18^O_HSL_ and lower, but variable, δ^18^O_ML_ values from 1200 to 1870 CE indicate relatively drier (low P/E) conditions. Superimposed on these long-term trends was a period of particularly wet conditions (high P/E) at Cahokia indicated by δ^18^O_HSL_ between 1000 and 1200 CE during the Medieval Climate Anomaly (MCA; 950–1250 CE). The MCA was followed by decreasing P/E (drier conditions) at Cahokia, with δ^18^O_HSL_ indicating an especially large reduction in P/E from 1200 to 1450 CE during the shift from the MCA to LIA (Fig. 2). During the MCA, high P/E corresponded with regional hydroclimate proxy evidence for higher water tables in Minnesota and Michigan^34^, reduced regional drought from the tree-ring Palmer Drought Severity Index^35–37^, warmer temperatures from a multi-site pollen-based summer temperature reconstruction^28^, and increased warm-season rainstorm events based on elevated lithic sediment delivery to Martin Lake^13^ (Fig. 3A). The opposite responses in the δ^18^O_HSL_ and δ^18^O_ML_ data and other proxy records during the onset of the LIA indicate prolonged regional drought from 1350 to 1450 CE. These MCA and LIA hydroclimate anomalies were associated with synoptic-scale changes in ocean-atmospheric circulation, with moisture from the Gulf of Mexico predominating during the MCA warm-season wet phase in response to − PNA-like and − PDO-like conditions, and Pacific and Arctic moisture predominating during the LIA warm-season drought in response to + PNA-like and + PDO-like conditions^13,15^. The most severe reduction in P/E (i.e., drought) between 1350 and 1450 CE was associated with particularly strong + PDO and + PNA conditions that resulted in a shift to winter-dominated precipitation (75%) and severe reduction in warm-season rainfall^13^.

Comparison of previously published archaeological data from Greater Cahokia and the American Bottom with the δ^18^O_HSL_ record suggests that mean state hydroclimate variations accompanied important cultural changes during the Mississippi period (1050–1450 CE)^39,40^. Previous research at Horseshoe Lake^20^ showed that lead (Pb) concentrations and total nitrogen isotopes (δ^15^N), interpreted as reflecting anthropogenic land-use disturbances, increased at Horseshoe Lake starting at ca. 1150 CE during the MCA when the δ^18^O_HSL_ record indicates increased P/E (Fig. 3). After 1200 CE, when the first fortifications were built around Cahokia’s central precinct^9^, a steady increase in δ^18^O_HSL_ indicates a reduction in P/E. The transition to drought conditions from ca. 1200 and 1400 CE correspond with a decline in Pb and δ^15^N at Horseshoe Lake (Fig. 3), as well as archaeological evidence for population decline and decentralization^41,42^, leading ultimately to the depopulation of Greater Cahokia^6,16,20^. The return of Pb and δ^15^N to background levels by 1350 CE is accompanied by a continued increase in δ^18^O_HSL_, suggesting the decline in human activity was associated with increasingly dry conditions at Horseshoe Lake (Fig. 3B, D).

The strong temporal correspondence between drought at Horseshoe and Martin lakes and the abandonment of Cahokia supports a causal relationship between regional climate and the decline of Greater Cahokia. One hypothesis is that increasing dependency on maize agriculture^17^ to support relatively high population densities (~ 21–27 persons km^−2^)^43^ and social complexity would have come with an increased susceptibility to climate change^16^. For example, empirical evidence has shown that maize yields are primarily determined by precipitation shortages during the growing season rather than excesses^21^. An extended period of dry conditions and low maize yields would have made it difficult to support the high population densities and level of social complexity that marked the growth and macro-regional influence of Cahokia between 1100 and 1200 CE^2^, when effective moisture and warm-season precipitation was high.

## Conclusions

The δ^18^O_HSL_ record indicates that Cahokia emerged as a regional center during the early Mississippi period (i.e., 1050–1200 CE) under relatively wet conditions with high effective moisture. These conditions likely contributed to the adoption of maize and development of a robust agricultural system that supported 15,000–20,000 individuals within and around Greater Cahokia between 1100 and 1200 CE. The onset of population declines within and around Cahokia ca. 1200 CE began as conditions trended toward lower effective moisture. Cahokia’s abandonment from 1350 to 1400 CE occurred during a prolonged regional drought, perhaps the most severe to strike the region in the last 1600 years. Paleoclimate records indicate that this drought was widespread in the midcontinental US and part of a general shift to more cold-season-like conditions during the LIA, with cooler, drier summers and colder, more severe winters in response to + PNA-like and + PDO-like conditions. These results support a strong link between climate anomalies and pre-Columbian demographic patterns in the American Bottom. Furthermore, these results indicate that the midcontinental US, presently a global agricultural center and home to more than 70 million people, is especially sensitive to severe and prolonged drought.

## Methods

### Sample collection, age modeling, δ^18^O, and δ^2^H analyses

Sediment cores and water samples were collected from Horseshoe Lake in 2012 and 2013 CE. The age-model and associated 95% confidence intervals for the interpolated ages were generated with CLAM (v. 2.2) using eight calibrated radiocarbon and 14 ^210^Pb dates^20^. The δ^18^O of authigenic calcite isolated from the Horseshoe Lake sediment archive was measured using a Thermo Gas Bench II connected under continuous flow to a Thermo MAT 252 stable isotope ratio mass spectrometer at Indianapolis University–Purdue University in Indianapolis (IUPUI). All lake sediment calcite δ^18^O results are reported in standard delta notation relative to Vienna Pee Dee Belemnite (VPDB) in ‰. Analytical precision, based on repeated measurements of standards, was 0.08 ‰ for calcite δ^18^O^20^. Regional surface water oxygen and hydrogen (δ^2^H) isotopes were analyzed at IUPUI using a Picarro L2130-i Analyzer^44^. Final values were corrected to Vienna Standard Mean Ocean Water (VSMOW) and are in ‰. Analytical precision was 0.02‰ and 0.30‰ for water δ^18^O and δ^2^H, respectively.

### Hydroclimate reconstructions

The oxygen isotopic composition of calcite precipitated from Horseshoe Lake’s water column reflects both variations in the isotopic composition of precipitation and the influence of local changes in the balance between precipitation and evaporation (P/E) variability on lake volume^45^. The oxygen isotopic composition of authigenic calcite can also be affected by variations in water temperature; however, estimated reconstructed temperature changes of +/− 2 °C are not large enough to explain the full range of δ^18^O_HSL_ variability observed in the Horseshoe Lake sediment record (up to 6.5‰). A 2 °C change during the LIA, for example, would then equate to a 0.72‰ change in δ^18^O in Horseshoe Lake (see “Discussion” section). Variations in precipitation amount and moisture sources can have a larger impact on the isotopic composition of regional lake water δ^18^O^13^. Changes in the sources of δ^18^O_precip_ therefore need to be accounted for in order to interpret the effects of P/E balance changes, and drought specifically, on δ^18^O_HSL_.

Regional δ^18^O_precip_ was characterized using the Martin Lake, IN, sediment calcite δ^18^O record (δ^18^O_ML_), which has been shown to reflect changes in moisture sources to the Midwest^13^ (Fig. 1). Average Martin Lake water δ^18^O plots at the intersection of the regional meteoric water line (RMWL) and the regional evaporation line (REL) along which Horseshoe Lake surface water evolves in response to evaporation (Fig. 1B). This indicates that Horseshoe Lake water evolved from an isotopic starting point indicated by Martin Lake δ^2^H–δ^18^O intercept along the RMWL. These isotopic relationships reflect the regional influence of synoptic-scale atmospheric circulation patterns that deliver precipitation from either northwesterly or southeasterly sources that have distinct δ^18^O signatures^13,26^. Back trajectory analysis of modern precipitation events together with climatological data spanning the last seven decades further demonstrates that moisture delivered to Martin Lake follows trajectories that pass over Horseshoe Lake (Fig. 1C, D). Therefore, comparisons of the δ^18^O_HSL_ and δ^18^O_ML_ records are used to investigate changes in P/E balance at Horseshoe Lake and the broader Midwest.

## Supplementary Information

Supplementary Information.
