# Supplementary material for: Severe Little Ice Age drought in the midcontinental United States during the Mississippian abandonment of Cahokia
Source: Sci Rep. 2021 Jul 5;11:13829. doi: 10.1038/s41598-021-92900-x (PMC8257696; doi:10.1038/s41598-021-92900-x)
Supplement: Supplementary file 1 — Supplementary Information. [file 41598_2021_92900_MOESM1_ESM.pdf]

*Supplemental Material for:*

# Severe Little Ice Age drought in the midcontinental United States during the Mississippian abandonment of Cahokia

**[1]David P. Pompeani*, [2]Broxton W. Bird, [3]Jeremy J. Wilson, [2]William P. Gilhooly III, [4]Aubrey L. Hillman, [5]Matthew S. Finkenbinder, [6]Mark B. Abbott**

*[1]Department of Geology, Kansas State University, Manhattan, KS 66506*
*[2]Department of Earth Science, Indiana University-Purdue University, Indianapolis, IN 46202*
*[3]Department of Anthropology, Indiana University-Purdue University, Indianapolis, IN 46202*
*[4]Department of Atmospheric and Environmental Sciences, University at Albany, State University of New York, Albany, NY 12222*
*[5]Department of Environmental Engineering and Earth Science, Wilkes University, Wilkes-Barre PA 18766*
*[6]Department of Geology and Environmental Science, University of Pittsburgh, Pittsburgh, PA 15260*

**corresponding author: dpompeani@ksu.edu*

## Impact of historical modifications to the Horseshoe Lake Watershed

The American Bottom floodplain where Horseshoe Lake is located has been extensively modified since the early 1900s. The modifications include the construction of levees and, most influential to Horseshoe Lake, rerouting of streams in the Horseshoe Lake watershed (i.e., Cahokia Creek). In order to gauge the impact these modifications may have had on Horseshoe Lake's hydrological mass balance, we quantitatively estimate residence times as the ratio of inflows ($m^3$ $yr^{-1}$) to lake volume ($m^3$).

### *Modern Inflows*

Groundwater is not considered because studies have shown that very little water in Horseshoe Lake is gained or lost via this pathway [1]. Horseshoe Lake currently receives inflows from two primary sources, 1) Cahokia Creek to the south and 2) the lake's immediate watershed to the north, which includes Nameoki Ditch, Elm South, and Granite City Steel effluent. The lake's immediate watershed provides 94,600 $m^3$ of water per day [1]. In the 1920's Cahokia Creek at Poag, IL, was diverted east to the Mississippi River. Stream gages were not established on Cahokia Creek between Poag and its confluence with Canteen Creek, so discharges were estimated by regressing drainage area against stream discharge using data from stream gauges on the American Bottom (Fig. S1). The local discharge-drainage area relationship ($r^2 = 0.99$, $p < 0.001$) indicates that Cahokia Creek discharges 103,200 $m^3$ of water per day (Table S1). Together with 41,200 $m^3$ of water per day from Canteen Creek (station 05589500), this equates

to 144,400 $m^3$ of water per day. Combined with 94,600 $m^3$ of water per day from the Horseshoe Lake's immediate watershed, this equates to 239,000 $m^3$ of water per day flowing into Horseshoe Lake. Over the course of a year, this comes to 87,235,000 $m^3$. Given an average depth of 1 m and a surface area of 9,712,500 $m^2$, Horseshoe Lake's volume today equals 9,712,500 $m^3$. As such, the 87,235,000 $m^3$ $yr^{-1}$ inflow is 9 times the volume of the lake today for an inflow to volume ratio of 9:1.

*Pre-modification Inflows*

Prior to the 1920s when Cahokia Creek's watershed was mainly intact, Cahokia Creek would have contributed 509,800 $m^3$ $day^{-1}$ (station 05588500). Together with Horseshoe Lake's other inflows, this equal 748,800 $m^3$ $day^{-1}$ or 273,312,000 $m^3$ $yr^{-1}$. During the Mississippian period, however, Horseshoe Lake was approximately 3 meters deep (based on dated sediment cores from the lake [2]). As such, Horseshoe Lake's volume during the Mississippian period was approximately 29,137,500 $m^3$ (or 3 m x 9,712,500 $m^2$). Given this volume, the inflow rate of 273,312,000 $m^3$ $yr^{-1}$ would be 9.4 times Horseshoe Lake's volume (9.4:1), which is essentially the same as the modern inflow to volume ratio of 9:1. This demonstrates that Horseshoe Lake's modern residence time is essentially the same as during the Mississippi Period and modern isotopic responses to changes in the ratio of precipitation to evaporation are analogous to those which would have occurred during the Mississippi Period. The transition to reduced inflows after the 1920's additionally corresponded to a doubling in sedimentation rates at Horseshoe Lake. This means that as inflows were decreasing because of Cahokia Creek's diversion at Poag, IL, the lake was infilling and losing accommodation space. As a result, Horseshoe Lake's hydrologic mass balance remained similar through time despite significant modifications in its watershed.

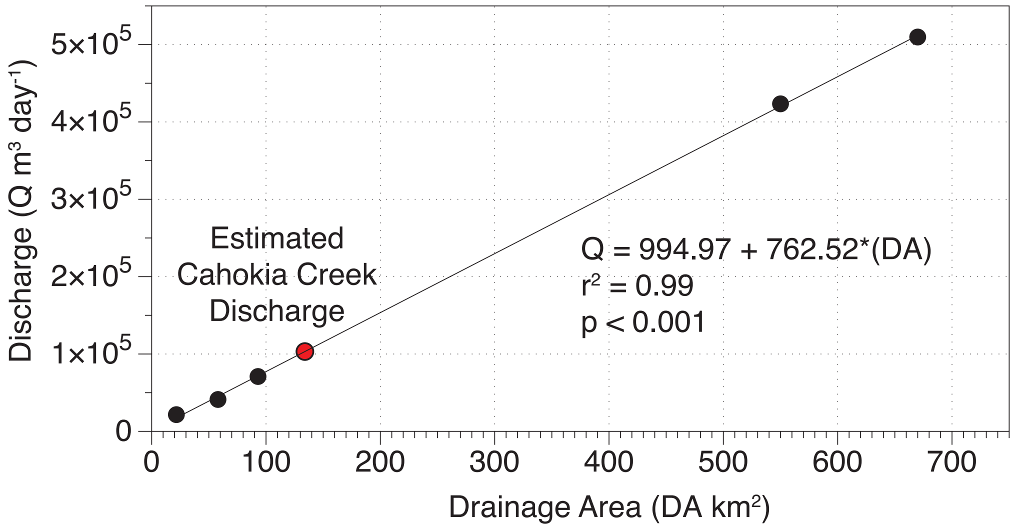

**Figure S1** Scatter plot of drainage area versus discharge for streams on the American Bottom either in or in close proximity to the Horseshoe Lake watershed. The estimated discharge for Cahokia Creek at Canteen Creek is shown with a red circle.

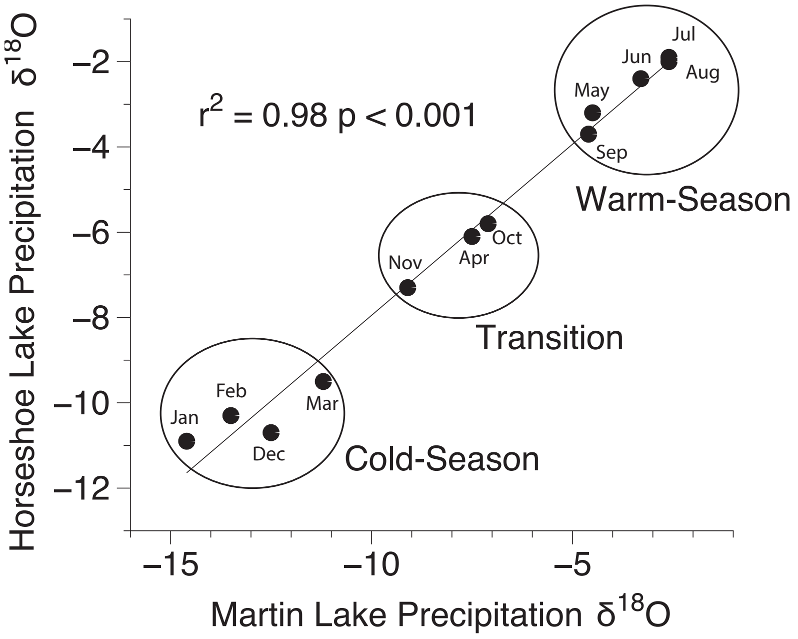


**Figure S2** Regression of monthly precipitation isotope data for Martin Lake, IN, and Horseshoe Lake, IL, showing similar isotopic variability related to changes in seasonal atmospheric circulation as shown in Figure 1 of the main text (data from waterisotopes.org). December through March is characterized by low isotopic values as a result of advection of moisture from Pacific and Arctic moisture sources. High isotopic values during the warm season from May through September reflect the advection of moisture from the Gulf of Mexico and Atlantic Ocean.

**Table S1** Drainage area and discharge for streams on the American Bottom.

| Drainage | Station ID | Length of Record | DA ($km^2$) | Discharge ($m^3$/day) |
|---|---|---|---|---|
| Judy's Branch at rte 157, IL | 5588720 | 2000-2011 | 21.6 | 21600 |
| Canteen Creek at Caseyville, IL | 5589500 | 1939-2982 | 58 | 41200 |
| Indian Creek at Wanda, IL | 5588000 | 1940-2021 | 93 | 70900 |
| Cahokia Creek at Edwardsville, IL | 5587900 | 1969-2021 | 550 | 423400 |
| Cahokia Creek Near Poag, IL | 5588500 | 1910-1912 | 670 | 509760 |
| *Cahokia Creek at Canteen Creek Estimated* | | | *134* | *103173* |


1 Hill, T. E., Evans, R. L. & Bell, J. S. Vol. 249 (Illinoise State Water Survey Contract Report Peoria, 1981).

2 Pompeani, D. P. *et al.* The environmental impact of a pre-Columbian city based on geochemical insights from lake sediment cores recovered near Cahokia. *Quaternary Research* **91**, 714-728, doi:10.1017/qua.2018.141 (2019).
